# Prevalence of abnormal birth weight and related factors in Northern region, Ghana

**DOI:** 10.1186/s12884-015-0790-y

**Published:** 2015-12-15

**Authors:** Abdulai Abubakari, Gisela Kynast-Wolf, Albrecht Jahn

**Affiliations:** Institute of Public Health, University Hospital, University of Heidelberg, Heidelberg, Germany; Community Nutrition Department, School of Allied Health Sciences, University for Development Studies, P.Box TL 1883, Tamale, Ghana

**Keywords:** Birth weight, Macrosomia, Low birth weight, Prevalence and malnutrition

## Abstract

**Background:**

Birth weight is a crucial determinant of the development potential of the newborn. Abnormal newborn weights are associated with negative effects on the health and survival of the baby and the mother. Therefore, this study was designed to determine the prevalence of abnormal birth weight and related factors in Northern region, Ghana.

**Methods:**

The study was a facility-based cross-sectional survey in five hospitals in Northern region, Ghana. These hospitals were selected based on the different socio-economic backgrounds of their clients. The data on birth weight and other factors were derived from hospital records.

**Results:**

It was observed that low birth weight is still highly prevalent (29.6 %), while macrosomia (10.5 %) is also increasingly becoming important. There were marginal differences in low birth weight observed across public hospitals but marked difference in low birth weight was observed in Cienfuegos Suglo Specialist Hospital (Private hospital) as compared to the public hospitals. The private hospital also had the highest prevalence of macrosomia (20.1 %). Parity (0–1) (*p* < 0.001), female gender (*p* < 0.001) and location (rural) (*p* < 0.001) were significantly associated with decreased risk of macrosomic births. On the other hand, female infant sex (*p* < 0.001), residential status (rural) (*p* < 0.001) and parity (0–1) (*p* < 0.001) were significantly associated with increased risk of low birth weigh.

**Conclusions:**

Our findings show that under nutrition (low birth weight) and over nutrition (macrosomia) coexist among infants at birth in Northern region reflecting the double burden of malnutrition phenomenon, which is currently being experienced by developing and transition counties. Both low birth weight and macrosomia are risk factors, which could contribute considerably to the current and future burden of diseases. This may overstretch the already fragile health system in Ghana. Therefore, it is prudent to recommend that policies aiming at reducing diet related diseases should focus on addressing malnutrition during pregnancy and early life.

## Background

Weight at birth can be classified into three categories, that is normal (birth weight ≥2.5 kg < 4.0 kg), too light (low birth weight (birth weight < 2.5 kg) or too heavy (macrosomia) (birth weight ≥ 4.0 kg). The last two conditions have adverse consequences on the life of the infant. Studies have shown that apart from short-term consequences such as high infant mortality and childhood growth failure among survivors, abnormal birth weight has a long-term risk in the form of high prevalence of adult coronary heart disease and type 2 diabetes [1]. According to Gluckman [2], this may be due to fetal or perinatal responses, which may include changes in metabolism, hormone production, and tissue sensitivity to hormones that may hinder the relative development of various organs, resulting in persistent changes in physiologic and metabolic homeostatic set points. Low birth weight (LBW) was also shown to have debilitating long-term consequences on childhood development, school achievement and adult capital, including achievement in height, economic productivity and birth weight of offspring [3, 4]. Macrosomia is seen as an important risk factor for prenatal asphyxia, death, and shoulder dystocia, and mothers of babies with macrosomia are at an increased risk of caesarean section, prolonged labor, postpartum hemorrhage, and perinatal trauma [5, 6].

Low birth weight is caused by either a short gestation period (<37 weeks) or retarded intrauterine growth (or a combination of both) [7]. Eleven percent (11 %) of all newborns in developing countries are born at term with low birth weight, a prevalence which is six times more than in developed countries [8, 9]. According to UNICEF [10] the prevalence of low birth weight babies in Ghana is 13.0 %.

Pre-term birth, maternal age (<20 years and >35 years), stress during pregnancy, maternal under nutrition before pregnancy and first parity may lead to low birth weight [11]. Other evidence adduced by Bategeka [12] show that factors such as low socio-economic status and use of services such as antenatal care and tetanus vaccination could influence birth weight.

On the other hand, macrosomia prevalence in the developed countries is between 5 % and 20 % but an increase of 15 %-25 % has been reported in the past decades, which is solely driven by an ascendance of maternal obesity and diabetes [6]. However, in developing countries data for the changing prevalence of macrosomia are scarce, in one study in China [13] researchers observed an increase from 6.0 % in 1994 to 7.8 % in 2005. In Ghana, there has been limited study on macrosomia but one recent study [14] reported 10.9 % macrosomic births among obese and overweight women in Bonso specialist hospital in Kumasi, Ghana. As the prevalence of diabetes and obesity in women of reproductive age increase in developing countries [15, 16], a corresponding increase in macrosomic births may be expected.

High pre-pregnancy weight or BMI, mother’s age (20-34years) and height, excessive gestational weight gain, gestational and pre-gestational diabetes mellitus, post-term pregnancy and male sex are found to be associated with macrosomia [17–19].

Ghana, like many other developing countries, is experiencing the double burden of malnutrition phenomenon where maternal under nutrition coexists with maternal over-nutrition [20, 21]. This is due to changes in life style, diet, urbanization, and reduced active commuting to work, use of energy saving devices and increasing sedentary employment that create ‘obesogenic’ environment [22].

Abnormal birth weight (low birth weight and macrosomia) may contribute to the current and future burden of chronic diseases. Complications during delivery as a result of macrosomia can lead to additional hazards to the mother and newborn in resource scarce settings as compared to resource rich settings because of the restricted availability of emergency obstetric and other essential care [23]. Therefore, the present study was aimed at contributing to understanding of the issues related to abnormal birth weight.

The aim of this study was to determine the prevalence of abnormal birth weight (low birth weight and macrosomia) and related factors such as socio-economic status and demographic characteristics of mothers in the Northern region of Ghana.

## Methods

The study examined the delivery records of the year 2013 in five hospitals in Northern Ghana where there has been steady economic growth in the past decade amidst extreme poverty, to determine the prevalence of abnormal birth weight. Findings from the study will provide useful information to policy makers for the design of appropriate public health interventions. The region is among the poorest regions in the country. The main occupation of the people in the region is agriculture and related activities. The region has 26 districts, with 24 of them being predominantly rural [24]. This notwithstanding, about half of the people live in urban areas with Tamale Metropolis, the regional capital being the most urbanized city in the region.

**Fig. 1:**
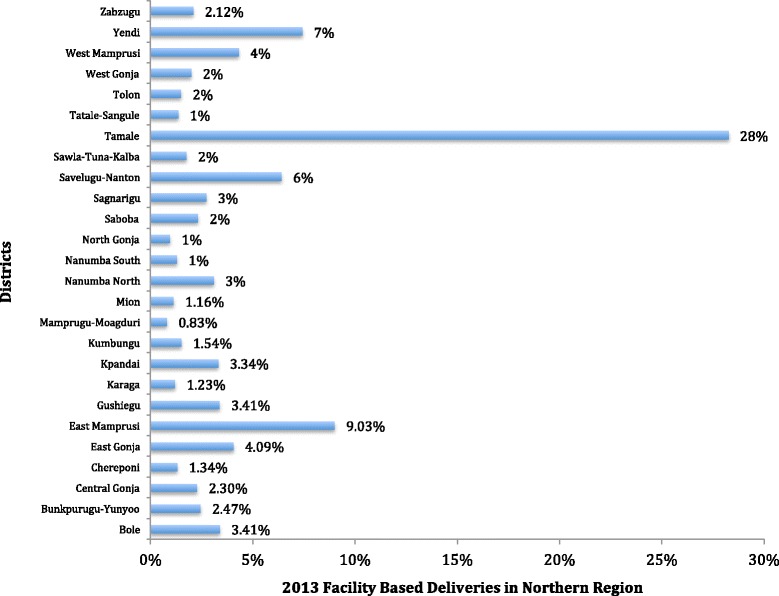
Pattern of facility-based deliveries in Northern Region

According to UNICEF [10] about 62.7 % deliveries took place in health facilities, but it was observed in one report that a greater proportion of facility-based deliveries usually occurred in urban areas [25]. As shown in Fig. 1, there are wide variations in the facility-based deliveries across districts in the region with three districts – Tamale, Sagnarigu and Savelegu-Nanton accounting for about 50 % of all the facility-based deliveries in the entire region.

Prior to the selection of health facilities for the study, ten key informants (midwives and programme planning, monitory and evaluation officers) who know and understand issues related to facility-based deliveries were identified and contacted. The discussion with these key informants was to get their inputs into the design and to seek their advice on the selection of the facilities. Their knowledge on the number of facility-based deliveries, availability and completeness of delivery records informed their recommendation. Based on this advice, five hospitals were purposively selected including one of the oldest private facilities in Tamale with the highest number of deliveries, Cienfuegos Suglo Specialist Hospital.

The five facilities were located in three districts, which accounted for about half of all the facility-based deliveries in 2013 in the region (Fig. 1). The hospitals are (1) Cienfuegos Suglo Specialist Hospital (CSSH) (2) Tamale Teaching Hospital (TTH) (3) Tamale Central Hospital (TCH) (4) Tamale West Hospital and (TWH) (5) Savelugu District Hospital (SDH). The Hospitals are located in the Tamale Metropolis, Sagnarigu District and Savelugu-Nanton District.

At each facility, the delivery register for 2013 was obtained and systematic random sampling was used to select individual records with a sampling probability of 4. In the case of Cienfuegos Suglo Specialist Hospital the number of deliveries was lower than in the public hospitals, so all the deliveries for 2013 were selected. The maternity records from each of the selected clients was obtained and transcribed onto a data collection form developed and pre-tested at the Yendi District Hospital. For each individual the following information was collected; age, parity, birth weight and Location of the mother. Individual records, which had birth weight missing, were not considered because it was the main dependent variable that was used in the analysis. Also, only singleton deliveries were used.

A low birth weight infant according to the world health Organization, is one born with birth weight <2.5 kg. Macrosomia was defined as birth weight greater than or equal to 4.0 kg. Therefore, this study considered all birth weights greater than or equal to 4.0 kg as macrosomic births. Birth weight ≥2.5 kg < 4.0 kg was considered normal.

The data was entered using Epi Info version 4.1 and transferred to Stata 12.1 for analysis. Summary statistics were computed to determine the general prevalence of abnormal birth weight and mean birth weight in the study population. Statistics were also computed to determine hospital specific prevalence and mean birth weight to assess socio- economic differences and heterogeneity of clients between the hospitals (rural, urban and peri-urban).

Standard of measurement across the hospitals was checked by placing a standard weight in the scale used to measure the newborn weight in all the hospitals; there was no significant difference observed in the five hospitals selected for this study.

A multinomial logistic regression model was used to determine the associations between maternal factors and abnormal birth weight. This model was used because the dependent variable in this study was nominal (Birth weight: 1 = Low birth weight, 2 = Normal, 3 = Macrosomia) and the Hausman test performed showed no evidence of violation of the independent of irrelevant alternative (IIA) condition. A *p*-value of less than 0.05 at 95 % confident level was considered as statistically significant.

Consistency and plausibility checks were done after the data entry to ensure that errors were reduced. Overall, 0.01 % of the data on birth weight, 0.05 % of the data on parity, 0.03 % of the data on maternal age were missing. Only 1100 (33 %) of the infants had their gestational age recorded and for that matter it was not used in the multinomial logistic regression analysis. No attempt was made to input missing data because there was no information about whetehr these data were missing at random [26].

Ethical approval for the research was obtained from the Navrongo Health Research Centre’s Ethical Review Board and Heidelberg University. Written permission was obtained from the health Hospitals to use the health records. However, because this study was done at the population level whereby data were extracted from medical records with no individual identifications, individual informed consent was not obtained.

## Results

### Characteristics of participating hospitals

The hospitals in this study offer various services to clients with varied socio-economic backgrounds. For instance, Cienfuegos Suglo Specialist Hospital (Private hospital) provides specialized obstetric and gynecology services to urban and high-income class in the Tamale Metropolis.. The clients of Tamale Teaching Hospital have mixed socio-economic backgrounds but are mostly from the high and middle-income classes who are largely urban dwellers. The hospital also gets clients from the lower income class who mostly come from the rural areas of the Metropolis and through referrals. Tamale Central Hospital also gets their clients predominantly from the middle-income class who are mostly urban dwellers but a significant proportion also comes from low-income class.

Tamale West Hospital provides Obstetric and Gynecology services to mostly the urban poor, some middle-income class and women from rural Tamale and surrounding districts while Savelugu District Hospital provides Obstetric and Gynecology services to mostly farming communities and a small proportion of middle-income class and urban dwellers in the District capital. Table 1 summarizes the characteristics of the selected hospitals.

**Table 1:** Characteristics of sample in the selected hospitals

Hospitals	Sample characteristics		
	Level	Location	Predominant socio-economic status of clients
CSSH	Secondary	Predominantly Urban	High income class
TTH	Tertiary	Predominantly Urban	High and Middle income class
TCH	Secondary	Urban/Peri-urban	Middle income class
TWH	Secondary	Urban/Rural	Middle and Low-income class
SDH	Secondary	Predominantly Rural	Low income class

Source: Key informant interview

### Birth weight and related factors

The data was analyzed for 3318 deliveries in 5 hospitals. The mean age of the mothers was 27 ± 6 years. The mean birth weight observed among the infants was 2.9 ± 0.73 kg. Prevalence of low birth weight was 29.6 % while the prevalence of macrosomia was 10.94 % (Table 2).

**Table 2:** Overall prevalence of abnormal birth weight and birth weight percentiles

Variables	n/N (%)
Low Birth weight	983/3318 (29.63)
Macrosomia	363/3318 (10.94)
Birth weight percentiles	Birth weight (kg)
5^th^	1.8
10^th^	2.0
50^th^	2.9
90^th^	4.0
95^th^	4.1

Data are presented in %(n/N) unless otherwise indicated

There were marginal differences observed in LBW prevalence between the public hospitals but a considerable differences were observed between the public hospitals and the private hospital ranging from 6.95 % in the Private Hospital to 39.13 % in the Tamale West Hospital (Table 3). However, there were large differences in the prevalence of macrosomia across all the hospitals ranging from 5.84 % in Tamale Central Hospital to 20.06 % in the Cienfuegos Suglo Specialist Hospital (Table 3). The proportion of normal birth weights was 72.99 %, 58.86 %, 72.99 %, 50.73 % and 52.33 % in CSSH, TTH, TCH, TWH and SDH respectively.

**Table 3:** Hospital specific prevalence of abnormal birth weight and birth weight percentiles

Hospitals	Birth weigh (kg)				Birth weight percentiles (kg)				
	N	Mean (SD)	Low Birth weight (<2.5 kg)	Macrosomia (≥4 kg)	5^th^	10th	50th	90th	95^th^
CSSH	349	3.2 (0.6)	23 (6.59 %)	70 (20.06 %)	2.4	2.6	3.2	4.1	4.1
TTH	1354	2.9 (0.80)	412 (30.43 %)	145 (10.71 %)	1.5	1.9	2.9	4.0	4.1
TCH	411	3.0 (0.52)	87 (21.17 %)	24 (5.84 %)	2.3	2.4	3	3.6	4.0
TWH	690	2.8 (0.72)	270 (39.13 %)	70 (10.14 %)	1.9	2.0	2.7	4.0	4.1
SDH	514	2.8 (0.72)	191 (37.16 %)	54 (10.51 %)	1.8	2.0	2.7	4.0	4.1

Data are presented in %(n/N), unless otherwise indicated

The risk of infants being born macrosomic as against normal in the Cienfuegos Suglo Specialist Hospital (clients are mostly from high income class) was 48 % higher as compared to those born in the Tamale Teaching Hospital (clients are mostly from high and middle income class but also gets referrals from across the socio-economic strata) whereas in Tamale Central Hospital (clients are mostly from middle income class) the risk of being born macrosomic versus normal was 58 % lower relative to Tamale Teaching Hospital when age, location and parity of the mother as well as sex of the infant were held constant (Table 4).

**Table 4:** Differences in abnormal birth weight in hospitals

Hospitals	Macrosomia		Low Birth weight	
	RRR (95 % CI)	*P*-value	RRR (95 % CI)	*P*-value
CSSH	1.48 (1.07-2.06)	0.018	0.21 (0.13-0.33)	<0.001
TTH	Reference		Reference	
TCH	0.42 (0.27-0.66)	<0.001	0.63 (0.48-0.82)	<0.001
TWH	1.06 (0.77-1.46)	0.712	1.60 (1.30-1.96)	<0.001
SDH	1.21 (0.85-1.72)	0.272	1.20 (0.92-1.45)	0.221

RRR = relative risk ratio. The estimates are based on maximum-likelihood multinomial logistic regression model controled for age of mother, Sex of baby, parity and location

On the other hand, the risk of infants being born LBW as against normal in the Cienfuegos Suglo Specialist Hospital was 79 % lower as compared to Tamale Teaching Hospital. In the same vein, the risk of babies being born LBW as against normal in Tamale Central Hospital was 37 % lower as compared to Tamale Teaching Hospital. However, the risk of babies being born LBW relative to normal in Tamale West Hospital (clients are mostly from low and middle income class) was 60 % higher compared to Tamale Teaching Hospital (Table 4).

More to the point, the risk of being born macrosomic for a first/second born as against normal was 52 % lower compared to a third/fourth born. Likewise, the risk of being born macrosomic as against normal for infants born by a rural mother was 45 % lower compared to an infant born by an urban mother. The risk of being born macrosomic as against normal for female infants was also 37 % lower compared to male infants (Table 5).

**Table 5:** Risk factors for abnormal birth weight adjusted for hospitals

Maternal and infant factors	Macrosomic Births	RRR	*P*-value	95 % Conf. Interval
Parity				
1	134/1800 (7.44 %)	0.48	<0.001	0.38-0.61
2-3	194/1277 (15.19 %)	Reference		
>4	35/241 (14.52 %)	1.09	0.655	0.73-1.66
Sex of baby				
Female	120/1448 (8.29 %)	0.63	<0.001	0.50-0.80
Male	220/1584 (13.89 %)	Reference		
Location				
Rural	45/787 (5.72 %)	0.55	<0001	0.39-0.78
Urban	309/2471 (12.51 %)	Reference		
Age of mother				
15-20	51/521 (9.79 %)	0.79	0.156	0.56-1.10
21-30	222/1920 (11.56 %)	Reference		
>30	90/877 (10.26 %)	0.84	0.200	0.64-1.10
	Low Birth weight			
Parity				
1	588/1800 (32.67 %)	1.33	<0.001	1.12-2.58
2-3	318/1277 (24.90 %)			
>4	77/241 (31.65 %)	1.24	0.179	0.90-1.72
Sex of baby				
Female	413/1584 (26.07 %)	1.52	<0.001	1.30-1.79
Male	516/1448 (35.64 %)	Reference		
Location				
Rural	363/787 (46.12 %)	2.15	<0.001	1.80-2.58
Urban	595/2471 (24.08)	Reference		
Age of mother				
15-20	143/521 (27.45 %)	0.82	0.091	0.65-1.03
21-30	582/1920 (30.31 %)			
>30	258/877 (29.42 %)	0.98	0.904	0.82-1.19
Normal		(base outcome)		

The final model was significant (Prob > chi2 = 0.0000)

Data are presented in %(n/N) unless otherwise indicated. RRR = relative risk ratio. The estimates are based on maximum-likelihood multinomial logistic regression models adjusted by Hospital of birth

Conversely, the risk of being born LBW for a first/second born relative to normal was 33 % higher compared to a third /fourth born. In the same vein, the risk of a female infant being born LBW relative to normal was 52 % higher compared to a male infant. Moreover, the risk of a rural woman given birth to a LBW baby as against normal was 2.15 times higher compared to an urban woman (Table 5).

## Discussion

This study is the first of its kind in Ghana that examines the prevalence of abnormal birth weight (low birth weight and macrosomic births). The findings of the study show that prevalence of low birth weight in Northern Ghana is high as compare to the national prevalence [10] and about three times higher than the WHO’s target of <10 %. These differences were expected since the Northern region is one of the poorest regions in Ghana [27] but not at this magnitude.

One of the factors that could explain this observation is that more of the regions’ population is engaged in subsistence farming which is mainly rain fed but due to regular drought and erratic rainfall patterns their harvest is not always sufficient to take them throughout the year. Quaye [28] reported that most farmer households in the northern region experience significant degree of food insecurity with food insecure periods spanning between 3 and 7 months. As a result there is high risk of maternal under nutrition in the region [29]. More so it is observed that there is a higher prevalence of malnutrition in Northern Ghana compared to the South [30]. This could be related to the high prevalence of low birth weight observed in this study.

The high prevalence of LBW observed in the present study could also be due to poor access to health services such as antenatal services due to socio economic barriers which are found to be strongly related to access to adequate prenatal services [12, 31]. About half of the population in the region lives in rural areas [24] and for that matter the residents have limited access to health services due to the deprived nature of their communities. They also lack access to social amenities such as electricity; good roads, potable water and as a result qualified midwives do not want to accept postings in to these communities.

Though it must be said that the government of Ghana has already put measures in place to improve access to pre-natal services by making antenatal care services free for all pregnant women across the nation, the success of these programs is limited due to socio-economic barriers. Other evidence adduced by Betegeka & co. [12] shows that low socio-economic status and use of services such as antenatal care services and tetanus vaccination could influence birth weight. Therefore, if the government policy were geared towards improving education of the girl child, which is very low in the North [24] as well as access to reproductive health and reduction of poverty in the area, it would play a crucial role towards enhancing the newborn birth weight in Northern Ghana.

The low prevalence of low birth weight in the private hospital was expected because it is obvious that the mothers who deliver in the private clinics are from high-income class of the society and have a better socio-economic status as compared to those who deliver in the public hospitals. Better socio-economic status is found to have a positive impact on delivery outcome. It is reported that improved maternal education, income and occupation have strong positive association with birth weight [32].

High prevalence of macrosomia was also observed in the present study but this appeared not to be very different from prevalence reported by studies in other developing countries, which used a similar cut-off point as this study especially in Africa. For example, a prevalence of 7.5 %, 8.4 % and 14.9 % was reported in Nigeria, Uganda and Algeria respectively [19] and in China investigators of one study revealed an increased macrosomic birth from 6.0 % in 1994 to 7.8 % in 2005 [13]. Another study in Port Harcourt in Nigeria shows macrosomia prevalence of 14.65 % [33] and in Ghana a recent study in one specialist hospital reported a prevalence of 10.9 % [14]. This is a clear manifestation of the double burden of malnutrition phenomenon, which is increasingly becoming a public health problem in developing countries where maternal under-nutrition coexists with maternal over-nutrition [20, 21]. Diabetes is believed to be an important risk factor for macrosomia [19] and could be a contributory factor for the increased prevalence of macrosomic births in Northern Ghana.

The study also confirmed established risk factors of macrosomia. For example, parity 2–3 was associated with increased risk of macrosomic births, which is consistent with the findings of the study conducted by Koyanagi & co. [19]. They showed that parity 2–3 or higher are associated with increased in odds ratios for macrosomia in developing countries. Beside this, female gender was also associated with increased risk of low birth weight, which also conforms to other findings. High risk of low birth weight was observed in infants born by mothers residing in rural areas and this was found to be consistent with findings of other studies. For example, studies conducted in United States [34] found high prevalence of low birth weight infants born by mothers in rural counties as compared to infants born by mothers in urban/large rural counties. The rural differences in birth weight could be due to a variety of factors such as poor access to health services [34–36] and socio-economic differences [37–40].

The study had some limitations because of irregularities of data in the delivery register. For example in every hospital the delivery records were under the responsibility of the nurse in charge of the maternity ward. They were kept in a variety of places and could only be located if the in-charge was present. There were difficulties in locating the maternity register in some facilities. This was because the active maternity register books were kept on the desk in the nursing station and the old one (previous years) kept by the maternity ward in-charge. Tamale Central Hospital could not provide delivery records for April, May, June, and July while at the Tamale West Hospital, January, February and March delivery records could not be found.

There was wide variation in the level and type of information provided in the maternity records. Some facilities recorded gestational age while others did not. The availability of data varied, ranging from 99.9 % for birth weight to 33 % for gestational age at delivery. As a result of the absense of gestational age at delivery in the delivery register in some of the hospitals gestational age, which is noted as a confounder of birth weight was excluded in the analysis.

## Conclusions

Our findings show that under nutrition (LBW) and over nutrition (macrosomia) coexist among infants at birth in the Northern region of Ghana reflecting the double burden of malnutrition phenomenon, which is currently being experienced by developing and transition counties. Given the paradox with increased risk of obesity at both ends of the birth weight spectrum and the potential health risks of low birth weight and macrosomia coupled with the ‘obesogenic’ environment that is being created by the steady urbanization in developing countries, there is a need for a paradigm shift in the fight against diet related diseases by focusing on preconception and maternal nutrition during pregnancy in a comprehensive and inclusive manner.

Our findings also provided empirical support for the link that existed between infant sex, maternal age, parity and differences in socio-economic classes and birth weight.

## Abbreviations

UNICEF: United Nations International Children’s and Emergency fund
WHO: World Health Organization
ACOG: American College of Obstetricians and Gynecologists
